# Microbial Community and Fermentation Characteristics of Native Grass Prepared Without or With Isolated Lactic Acid Bacteria on the Mongolian Plateau

**DOI:** 10.3389/fmicb.2021.731770

**Published:** 2021-10-01

**Authors:** Sihan You, Shuai Du, Gentu Ge, Tao Wan, Yushan Jia

**Affiliations:** ^1^Key Laboratory of Forage Cultivation, Processing and High Efficient Utilization, Ministry of Agriculture, Key Laboratory of Grassland Resources, Ministry of Education, College of Grassland, Resources and Environment, Inner Mongolia Agricultural University, Hohhot, China; ^2^National Engineering Laboratory of Biological Feed Safety and Pollution Prevention and Control, Key Laboratory of Molecular Nutrition, Ministry of Education, Key Laboratory of Animal Nutrition and Feed, Ministry of Agriculture and Rural Affairs, Key Laboratory of Animal Nutrition and Feed Science of Zhejiang Province, Institute of Feed Science, Zhejiang University, Hangzhou, China

**Keywords:** isolation, lactic acid bacteria, bacterial community, native grass, fermentation quality

## Abstract

This study aimed to isolate and identify lactic acid bacteria (LAB) from the native grass and naturally fermented silage from the Mongolian Plateau. The effect of selected strains on bacterial community and quality of native grass silage was also studied. Strains XM2, 265, and 842 could grow normally at 15°C–30°C, pH 4.0–8.0, and NaCl 3 and 6.5%; they were identified as *Lactiplantibacillus plantarum* subsp. *plantarum*, *Pediococcus acidilactici*, and *Latilactobacillus graminis*, by sequencing 16S rRNA, respectively. The three strains (XM2, 265, and 842) and one commercial additive (L) were used as inoculants and singularly added to the native grass. Compared to the control, the dry matter content was significantly (*p* < 0.05) lower in L and XM2 groups. The water-soluble carbohydrate content was significantly (*p* < 0.05) higher in control than in other groups. Compared with the control, the crude protein and ammonia nitrogen contents were significantly (*p* < 0.05) higher and lower in the LAB-treated groups, and the acid and detergent fiber contents were significantly (*p* < 0.05) reduced in the L and XM2 groups than those in other groups. There was a significant (*p* < 0.05) difference in the pH value, lactic acid content, and lactic acid-to-acetic acid ratio in L and XM2 groups than in other groups. Compared with the control, the number of LAB was significantly (*p* < 0.05) higher in LAB-treated silages, whereas no significant (*p* > 0.05) differences were observed in yeast and aerobic bacteria in all groups. Compared to the control, the Shannon index was significantly (*p* < 0.05) reduced. Simpson and Chao1 were significantly (*p* < 0.05) increased. Principal coordinate analysis based on the unweighted UniFrac distance showed clear separation of the bacterial community in fresh materials and LAB-treated silages. Besides, compared to the control, the principal coordinate analysis of LAB-treated silages was also separate. After 30 days of fermentation, the relative abundance of *Firmicutes* increased and was the primary phylum in all silages. Compared with the control, the abundance of *Firmicutes* and *Proteobacteria* was significantly (*p* < 0.05) higher and lower in L and XM2 groups. In contrast, no significant differences were observed among control, 265, and 842 groups. At the genus level, the relative abundance of *Lactobacillus*, *Enterobacter*, *Pediococcus*, and *Weissella* was increased and dominated the native grass fermentation. Compared with the control, the abundance of *Lactobacillus* was significantly (*p* < 0.05) higher in L, XM2, and 842 groups, while no significant (*p* > 0.05) differences were observed between the control and 265 groups. The abundance of *Pediococcus* was higher than that in other groups. Consequently, the results demonstrated that LAB significantly influenced silage fermentation by reconstructing microbiota, and *Lactobacillus* was the dominant genus in the native grass silages. Furthermore, the results showed that strain XM2 could effectively improve the silage quality, and it is considered a potential starter for the native grass silage.

## Introduction

Native grasslands are widely distributed in the north and west of China, including the Mongolian Plateau, Qinghai, and the Tibet Plateau, are an essential resource in animal production, and grow well in fall and autumn, providing sufficient nutrition and biomass for animals (Du et al., 2019). Hay is a traditional method for maintaining forage, while the disadvantages of native grass hay, including hay quality and palatability, make it hard to shake off the seasonal and yearly imbalance of available forage (Yan et al., 2019). Ensiling is a traditional way for preservation of animal feed and green forage crops because it can supply forages for animals year-round, effectively reduce the nutrition loss of forages, and prolong storage time (Pahlow et al., 2003). However, the moisture and water-soluble carbohydrate (WSC) content and the number of lactic acid bacteria (LAB) were lower than the requirement for a well-preserved silage (You et al., 2021). Therefore, it is difficult to produce high-quality silage of native grass using natural fermentation.

Generally, LAB additives are a practical method for improving fermentation quality, which is widely used for silage preparation. The LAB group has been selected from various forages, including alfalfa (Ogunade et al., 2018), *Elymus nutans* (Xu et al., 2018), oat (Romero et al., 2017), King grass (Shah et al., 2018), *Moringa oleifera* (Wang Y. et al., 2018), native grass (You et al., 2021), and Teff (Tilahun et al., 2018). Ensiling involves complex microbial interactions, and the microbial ecology associated with silages was conducted using classical microbial techniques (Keshri et al., 2019). Studies on the microbial composition during silage fermentation have shown that *Lactobacillus* often plays an important role during the later stages of ensiling and manufactures the amount of lactic acid to improve silage fermentation (Ding et al., 2019). Previous studies also found that *Lactobacillus*, *Weissella*, *Pseudomonas*, and *Leuconostoc* spp. in silage samples were the dominant genera (Guan et al., 2018; Keshri et al., 2019; Yang et al., 2019; Bai et al., 2020). Although the isolation, selection, and application of LAB are critical for silage fermentation and bacterial communities, the LAB was isolated from various forages and grasses with different effects on silage fermentation by various environments (Yang et al., 2010; Zhang et al., 2015; Yan et al., 2019), but few LAB have been isolated and applied on native grass in the Mongolian Plateau. Some LAB strains were isolated from the native grass and naturally fermented silage in the Mongolian Plateau in our research. These strains grew under low-pH conditions in an anaerobic environment, producing more lactic acid, and widely used carbohydrates. Still, the effects of these strains on silage fermentation of native grass remain unclear.

Consequently, this study aimed to select and identify LAB strains using physiological and morphological tests and molecular methods from native grass, and its effects on the bacterial community and the silage quality of native grass were also studied.

## Materials and Methods

### Lactic Acid Bacteria Strains

A total of 23 LAB strains were isolated from 35 native grass and naturally fermented silage samples. The grass was harvested at the milk stage from typical and meadow steppe in Inner Mongolia Plateau, China, in August 2018 and 2019. The grassland contained typical and meadow steppe flora of HulunBuir, Inner Mongolia, with the Giant Feathergrass (*Stipa gigantea* Link.) and Chinese Leymus (*Leymus chinensis* [Trin.] Tzvel.), and the Baical Needlegrass (*Stipa baicalensis* Roshev.) and Chinese Leymus (*Leymus chinensis* [Trin.] Tzvel.) being the dominant species, respectively. The 10-g silage samples from each silage sample were homogenized with 90 ml of distilled water; serial dilutions were used for potential LAB culturing and purification by streaking on de Man, Rogosa, Sharpe (MRS) agar (Difco Laboratories, Detroit, MI, United States) four times at 30°C for 48 h and stored at −80°C in MRS broth with 20% glycerol.

### Physiological and Morphological Tests

Gram staining, catalase activity, and gas production from glucose were determined as previously reported (Duan et al., 2008; You et al., 2021). Growth at different temperatures and pH environments (HCl or NaOH were used for adjusting pH) was based on the method of Cai et al. (1999c). Salt tolerance was determined using the method of Wang S. et al. (2018). The growth was measured and evaluated using optical density (OD) value (You et al., 2021). API 50 CH (BioMérieux, Marcy l’ Etoile, Lyons, France) was used to determine the carbohydrate assimilation (Cai et al., 1999a).

### Lactic Acid Bacteria Identification by 16S rRNA Sequencing

To extract the DNA of the screened strains, Bacterial DNA kit was used (Tiangen Biotech Co., Ltd., Beijing, China). The primers 27F (5′-AGAGTTTGATCCTGGCTCAG-3′) and 1492R (5′-TACGGCTACCTTGTTACGACT-3′) were used in the polymerase chain reaction (PCR) (Cai et al., 1999b). The species identification was conducted as previously reported (Zhang et al., 2015). The 16S rRNA sequences were identified using BLAST analysis on GenBank (Ennahar et al., 2003).

### Preparation of Silage

Native grass was harvested at the milk stage on August 20, 2020, in Chifeng, China. The native grassland was meadow steppe flora of Baarin Left Banner, Inner Mongolian Plateau, including Giant Feather Grass (*S. gigantea* Link.), Chinese Leymus [*L. chinensis* (Trin.) Tzvel.], and Dahurian Bushclover [*Lespedeza davurica* (Laxm.) Schindl] as the dominant species. Strains XM2, 265, and 842, and commercial LAB additive (defined as L, CH, *Lactobacillus plantarum*, Snow Brand Seed Co., Ltd, Sapporo, Japan) were used as inoculants. All LAB strains were added singularly at 10^5^ cfu/g fresh materials (FM), and the control was added to the same volume of distilled water (Wang Y. et al., 2018; You et al., 2021). The FM was chopped using a manual forage chopper (Xianglong Co., Ltd., Linyi, China) with 2- to 3-cm lengths and was immediately moved to the laboratory. About 500 g of FM in a plastic polyethylene bottle (1 L capacity) and air were eliminated. Each treatment was prepared with three replicates and stored at ambient temperature for 30 days.

### Chemical and Fermentation Characteristics Analysis

Dry matter content of raw material and 30 days of fermentation of native grass silage samples was determined by drying samples in a forced-air oven at 65°C for 72 h. However, based on the Association of Official Analytical Chemists, crude protein (CP) content was determined (Association of Official Analytical Chemistry, 1990). Fiber fractions were determined according to the method of Van Soest et al. (1991). The WSC content was determined as previously reported (Murphy, 1958).

The 10-g sample was added to 90 ml of distilled water and remained for 24 h at 4°C in a refrigerator, and the extract was filtered through four layers of cheesecloth. The pH value and organic acid contents were determined using the method of You et al. (2021). The ammonia nitrogen (NH_3_-N) content was assessed as described by Kleinschmit et al. (2005).

### Microbial Composition Analysis

The microbial compositions of FM and silages were analyzed using the plate count method. Under anaerobic conditions on MRS agar incubated at 30°C, the counts of LAB were quantified for 48 h (You et al., 2021); coliform bacteria, aerobic bacteria, mold, and yeast were calculated using the previously reported method (You et al., 2021) and expressed on colony-forming units (cfu)/g of FM.

### DNA Extraction, Polymerase Chain Reaction Amplification, and Sequencing

Bacterial community genomic DNA was extracted from native grass and silage samples using the E.Z.N.A.^®^ sample DNA kit (Omega Bio-tek, Norcross, GA, United States). The NanoDrop 2000 UV-vis Spectrophotometer (Thermo Scientific, Wilmington, United States) was used in determining the concentration and purity of the extracted DNA and 1% agarose gel electrophoresis was used to analyze the quality of extracted DNA (Bai et al., 2020). All extracted DNA samples were frozen at −20°C for analysis.

Majorbio Bio-Pharm Technology Co., Ltd. (Shanghai, China) performed PCR amplification and bioinformatic analysis. The hypervariable region V3–V4 of the bacterial 16S rRNA gene was amplified using primer 338F (5′-ACTCCTACGGGAGG CAGCAG-3′) and 806R (5′-GGACTACHVGGGTWTCTAAT-3′). The 16S amplification was conducted according to the description of Tian et al. (2017). Raw sequence data were uploaded to the NCBI’s Sequence Read Archive under study accession number PRJNA758799.

### Statistical Analyses

Paired-end reads were assigned to samples based on their unique barcode, truncated by cutting off the barcode and primer sequence and merged using FLASH (v1.2.8) (Mago and Salzberg, 2011). Under specific filtering conditions, quality filtering on raw tags was performed to obtain high-quality clean tags according to fqtrim (v0.94) (Li et al., 2021). The chimeric sequences were filtered using Vsearch software (v2.3.4) (Rognes et al., 2016), and the high-quality sequences with ≥ 97% similarity were assigned to the same operational taxonomic units (OTUs) (Li et al., 2021). Taxonomic summaries were conducted by calculating the relative abundance across samples and normalizing to 100%. Alpha diversity and beta diversity were computed by normalizing to the same sequences randomly using QIIME2. OmicStudio tools^1^ were used to perform most of the graphics drawing. The chemical composition, silage quality, and microorganism population of silages were performed using a one-way analysis of variance with three replicates. Duncan’s tests separated significant differences and were considered statistically significant at the 5% probability level (SAS Inc, 2007).

## Results

### The Selection of Isolated Lactic Acid Bacteria Based on Morphological and Physiological Tests

The selection of isolated LAB based on morphological and physiological tests is indicated in Table 1. All isolated strains were Gram-positive and catalase-negative, the XM2 strain was rod-shaped, and strains 265 and 842 were cocci-shaped. All strains normally grew at 15 and 30°C, pH 4.0–8.0, while strain 265 normally grew at 50°C, and strains XM2 and 842 grew normally at 10°C. Under NaCl concentrations of 3.0 and 6.5%, all isolated strains grew normally. According to the results of gas for glucose, the three strains possessed similar fermentation patterns and were homofermentative, while different ferment carbohydrates are indicated in Table 2. The three isolated strains that could ferment carbohydrates in API are listed in Table 2. In this study, the GenBank data library was used to analyze the similarity through BLAST^2^, strain XM2 possessed a higher similarity with *L. plantarum*, strain 265 possessed a higher similarity with *Pediococcus acidilactici*, and strain 842 possessed a higher similarity with *Lactobacillus graminis*, with 99.87, 99.58, and 99.63% similarities in their 16S rRNA gene sequence, respectively (Table 3 and Supplementary Figure 1). The strains’ nucleotide sequences were also transferred to GenBank with accession numbers MT358326, MT358327, and MT358333 for XM2, 265, and 842, respectively. Based on the new classification of *Lactobacillus* genus, strains XM2, 265, and 842 were identified as *Lactiplantibacillus plantarum* subsp. *plantarum*, *P. acidilactici*, and *Latilactobacillus graminis*, respectively.

**TABLE 1:** The selection of isolated lactic acid bacteria on the base of the morphological and physiological tests.

Items	XM2	265	842
Shape	Rod	Cocci	Cocci
Gram stain	+	+	+
Gas for glucose	−	−	−
Catalase	−	−	−
Fermentation type	Homo	Homo	Homo
**Growth at temperature (°C)**			
5	w	−	w
10	+	−	+
15	+	+	+
30	+	+	+
45	w	+	+
50	w	+	w
**Growth at pH**			
3.0	w	−	−
3.5	+	+	−
4.0	+	+	+
5.0	+	+	+
6.0	+	+	+
7.0	+	+	+
8.0	+	+	+
**Growth in NaCl (%)**			
3.0	+	+	+
6.5	+	+	+

*+, positive;−, negative; w, weakly positive. Homo; homofermentative.*

**TABLE 2:** The characteristics of isolated lactic acid bacteria on the base of carbohydrate fermentation.

Item	XM2	265	842
L-Arabinose	−	−	+
Ribose	+	+	+
D-Xylose	−	+	−
D-Galactose	+	+	+
D-Glucose	+	+	+
D-Fructose	+	+	+
D-Mannose	+	+	+
D-Mannitol	+	−	−
D-Sorbitol	+	−	−
Methyl-αD-Mannopyranoside	+	−	−
N-Acetyl Glucosamine	w	w	+
Amygdalin	+	w	w
Arbutin	w	w	−
Esculin	+	+	+
Salicin	w	w	w
Cellobiose	+	+	+
Maltose	+	−	−
Lactose	+	−	−
Melibiose	+	−	−
Sucrose	+	−	−
Trehalose	+	+	+
Melezitose	+	−	−
Raffinose	+	−	−
Gentiobiose	w	w	w
D-Tagatose	−	w	−
D-Arabitol	w	−	−
Gluconate	w	−	−

*All strains gave negative results for glycerol, erythritol, D-arabinose, L-xylose, D-adonitol, methyl-βD-xylopyranoside, L-sorbose, L-rhamnose, dulcitol, inositol, methyl-αD-glucopyranoside, inulin, starch, glycogen, xylitol, D-turanose, D-lyxose, D-fucose, L-fucose, L-arabitol, 2-keto-gluconate, and 5-keto-gluconate. +, positive; -, negative; w, weakly positive.*

**TABLE 3:** The results of isolated lactic acid bacteria on the base of 16S rRNA gene sequences.

Strain	Accession number	16S rRNA gene sequencing data (closest relative)	Similarity (%)
XM2	NR_115605.1	*Lactobacillus plantarum* JCM 1149	99.87
265	NR_042057.1	*Pediococcus acidilactici* DSM 20284	99.58
842	NR_042438.1	*Lactobacillus graminis* G90	99.63

*Chemical composition and microbial population of native grass prior to ensiling.*

### The Chemical Compositions and Microbial Population of the Fresh Materials Before Ensiling

The chemical compositions and microbial population of the substrates prior to ensiling are shown in Table 4. The moisture content of native grass was 40.66%, and WSC, CP, NDF, and ADF were 4.36, 11.74, 58.15, and 30.07% on a DM basis, respectively. Microbial populations in the native grass for LAB, coliform bacteria, aerobic bacteria, and yeasts were 4.01, 6.93, 7.68, and 7.41 log_1__0_cfu/g FM, respectively. Mold was not detected in native grass.

**TABLE 4:** Chemical composition and microbial population of native grass prior to ensiling.

Items	Native grass
Dry matter (%)	59.34
Water-soluble carbohydrates (% DM)	4.36
Crude protein (% DM)	11.74
Acid detergent fiber (% DM)	30.07
Neutral detergent fiber (% DM)	58.15
Lactic acid bacteria (log_10_ cfu/g FM)	4.01
Yeast (log_10_ cfu/g FM)	7.41
Aerobic bacteria (log_10_ cfu/g FM)	7.68
Coliform bacteria (log_10_ cfu/g FM)	6.93
Mold (log_10_ cfu/g FM)	ND

*DM, dry matter; FM, fresh matter; cfu, colony-forming units; ND, not detected.*

### Chemical Compositions, Silage Quality, and Microbial Populations of Ensiling

The chemical compositions, silage quality, and microbial populations on 30 days of native grass ensiling are indicated in Table 5. There were significant (*p* < 0.05) differences in DM contents in the L and XM2 groups than in the control, while no significant (*p* > 0.05) differences were observed among control, 265, and 842 groups. The WSC content was significantly (*p* < 0.05) greater in control than in other groups. Compared to control, the NH_3_-N and CP contents were significantly (*p* < 0.05) greater and lower than in the L and XM2 groups, respectively. The NDF and ADF contents also significantly decreased in L and XM2 groups compared to that in the control. After 30 days of fermentation, compared to control, the pH in inoculated silages was significantly (*p* < 0.05) lower, and lactic acid (LA) content and lactic acid-to-acetic acid ratio were significantly (*p* < 0.05) increased. The number of LAB was significantly (*p* < 0.05) greater in inoculated silages than in control. No significant (*p* > 0.05) differences were observed in yeast and aerobic bacteria in all groups, and coliform bacteria and mold were lower than detectable in all groups.

**TABLE 5:** Chemical compositions, fermentation characteristics, and microbial populations on 30 days of ensiling.

Item	CON	L	XM2	265	842	SEM	*p*-value
Dry matter (%)	58.69a	56.84b	56.61b	57.77ab	57.80ab	0.247	0.0195
WSC (g/kg)	3.34a	2.29cd	2.07d	2.36c	2.71b	0.123	<0.0001
Crude protein (% DM)	9.58d	10.36b	10.73a	10.04c	9.85c	0.110	<0.0001
NH_3_-N (g/kg)	6.28a	5.02c	4.56c	5.52b	5.52b	0.163	<0.0001
Acid detergent fiber (% DM)	33.29a	31.61bc	31.11c	32.60ab	32.26abc	0.243	0.0114
Neutral detergent fiber (% DM)	59.27a	56.97bc	56.11c	58.68a	58.10ab	0.344	0.0019
pH	4.42a	4.10cd	4.05d	4.17bc	4.21b	0.035	<0.0001
Lactic acid (g/kg)	13.10c	21.62a	21.49a	15.46b	12.88c	1.060	<0.0001
Acetic acid (g/kg)	2.86ab	2.62b	2.38b	2.62b	3.57a	0.147	0.0677
Propionic acid (g/kg)	0.22b	0.32a	0.27ab	0.27ab	0.20b	0.016	0.0913
lactic to acidic acid ratio	4.58bc	8.41a	9.17a	6.01b	3.67c	0.614	0.0003
Lactic acid bacteria (log_10_ cfu/g FM)	7.02c	7.50b	7.75a	7.42b	7.35b	0.048	<0.0001
Yeast (log_10_ cfu/g FM)	2.89	2.45	2.58	3.11	2.17	0.191	0.5716
Aerobic bacteria (log_10_ cfu/g FM)	3.88	3.89	3.69	3.91	3.84	0.035	0.2711
Coliform bacteria (log_10_ cfu/g FM)	ND	ND	ND	ND	ND		
Mold (log_10_ cfu/g FM)	ND	ND	ND	ND	ND		

*DM, dry matter; FM, fresh matter; cfu, colony-forming units; ND, not detected; CON, control group; L, commercial inoculant group; XM2, strain XM2 group; 265, strain 265 group; 842, strain 842 group. SEM, the error of the means. a–c Means within a column without a common superscript letter difference, at p < 0.05 level.*

### Microbial Diversity of Fresh Materials and Native Grass After Ensiling

This study also analyzed the bacterial microbiota in silages and identified the species-discriminatory taxa. The sequencing information and bacterial diversity analysis are indicated in Supplementary Table 1. Compared to FM, the OTUs, Shannon, and Chao1 decreased significantly (*p* < 0.05). The Good’s coverage of all groups was more than 99%. Compared to the control, the Shannon index was significantly (*p* < 0.05) reduced, and Simpson and Chao1 were significantly (*p* < 0.05) increased.

**FIGURE 1:**
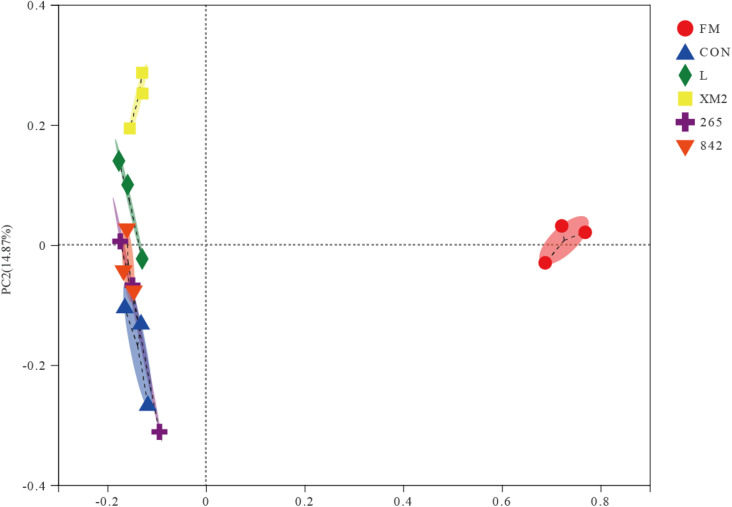
Principal coordinate analysis (PCoA) of the bacterial community of FM and native grass on 30 days of ensiling. FM, fresh native grass; CON, control group; L, commercial inoculant group; XM2, strain XM2 group; 265, strain 265 group; 842, strain 842 group.

Next, principal coordinate analysis (PCoA) was conducted based on the unweighted UniFrac distance to determine whether the microbial community structure changed in FM and silages (Figure 1). The PCoA plot exhibited a clear separation of bacterial community in FM and silages; besides, L, XM2, and other LAB-treated groups were also separate.

**FIGURE 2:**
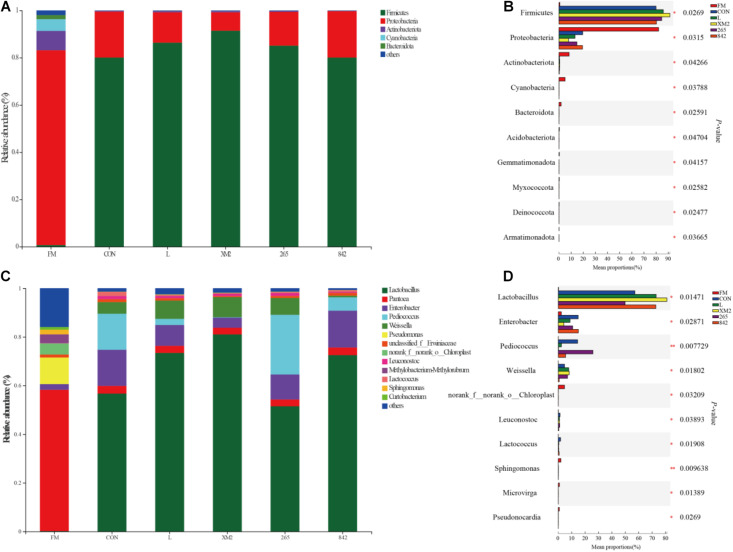
The bacterial community of FM and native grass on 30 days of ensiling. **(A)** The bacterial community was shown at the phylum level. **(B)** The extended error bar plot displaying the significant differences among groups at the phylum level **(C)**. The bacterial community was shown at the genus level. **(D)** The extended error bar plot displaying significant differences among groups at the genus level. *Shows that the significant difference was at *p* < 0.05 level. FM, fresh native grass; CON, control group; L, commercial inoculant group; XM2, strain XM2 group; 265, strain 265 group; 842, strain 842 group.

The relative abundance of bacteria in FM and silages of 30 days of ensiling are indicated in Figure 2. In FM, the dominant phyla of native grass were *Proteobacteria* and *Actinobacteriota* (Figure 2A). After 30 days of ensiling, the relative abundance of *Firmicutes* increased and was the primary phylum in all silages (80%, Figure 2B), and significant (*p* < 0.05) differences were observed among FM and silages in *Firmicutes*, *Proteobacteria*, and *Actinobacteriota* at the phylum level (Figure 2B). Compared to control, the abundance of *Firmicutes* and *Proteobacteria* was significantly (*p* < 0.05) higher and lower in L and XM2 groups, whereas no significant differences were observed among control, 265, and 842 groups. In FM, the dominant phyla of native grass were *Pantoea*, whose abundance was more than 50% (Figure 2C). After 30 days of fermentation, the relative abundance of *Lactobacillus*, *Enterobacter*, *Pediococcus*, and *Weissella* was increased and dominated the native grass fermentation among silages (80%, Figure 2B), and significant (*p* < 0.05) differences were observed among FM and silages in *Lactobacillus*, *Enterobacter*, *Pediococcus*, and *Weissella* (Figure 2D). Compared to control, the abundance of *Lactobacillus* was significantly (*p* < 0.05) higher in L, XM2, and 842 groups, while no significant (*p* > 0.05) difference was observed between control and 265 groups. The abundance of *Pediococcus* was the (*p* < 0.05) highest than in other groups.

**FIGURE 3:**
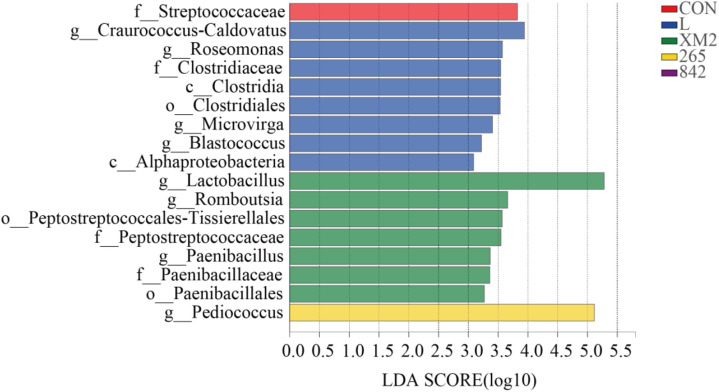
The linear discrimination analysis (LDA) coupled on the bacterial community of native grass on 30 days of ensiling with effect size (LEfSe) analysis. The significant difference in species was estimated by an LDA score greater at default score = 3. The length of the histogram shows the LDA score of differences in these groups. CON, control group; L, commercial inoculant group; XM2, strain XM2 group; 265, strain 265 group; 842, strain 842 group.

The linear discriminant analysis effect size (LefSe) was applied to explore the relative richness (*p* < 0.05, LDA > 3.0) of silages (Figure 3). The *Lactobacillus* was enriched in the XM2 group, and *Pediococcus* was enriched in the 265 group.

## Discussion

Forage crops, silages, and dairy products have been found in various LAB species, and many isolates have been identified as the *Lactobacillus* group (You et al., 2021). Previously published studies also showed that *Lactobacillus* is the primary microorganism on forages and silages (Cai et al., 1999c). However, it is challenging to identify the species among differentiated species by morphological, physiological, and biochemical tests (Chen et al., 2013). The 16S rDNA sequence analysis identifies organisms by genus and species (Ennahar et al., 2003).

Similarly, strains XM2, 265, and 842 at pH 4.0 could grow normally and strain XM2 weakly grew at pH 3.0. These results showed that strain XM2 had a high tolerance to acidity and the ability to grow and thrive in low-pH environments, which agree with the findings that *L. plantarum* displayed high resistance to a low-pH environment (Wang S. et al., 2018). Additionally, these isolated strains grew normally from 5°C to 30°C. While significant differences were observed among these strains, strains XM2 and 842 grew normally at 10°C, and strain 867 grew well at 50°C, which could be attributed to the unique environment, long periods of natural selection, and evolution on the Inner Mongolian Plateau (You et al., 2021). Therefore, the unique traits of these isolated LAB strains could have applications as additives on silage fermentation. What is more, strain XM2 could ferment more substrates than the other strains, which is consistent with the previous report that shows that the *L. plantarum* group could ferment a wide variety of carbohydrates (McDonald et al., 1991).

The moisture content of the grass is one of the essential factors that can directly influence the silage fermentation quality. In this study, the moisture content of native grass agrees with the previous report that shows the range of 44.93–47.51% in meadow steppe on the Mongolian Plateau (Hou et al., 2017). The plant diversity and environment may contribute to the difference. The WSC content was lower than an adequate WSC concentration that good silage needs WSC content higher than 5% on DM for LAB fermentation (Amer et al., 2012). This result follows the previous study on the low WSC content in native grass (Hou et al., 2017). The CP, ADF, and NDF contents followed those of Du et al. (2019), who discovered the native grass with a lower CP content and higher NDF and ADF contents.

Generally, the silage fermentation process and fermentation quality were determined by the counts and species of epiphytic LAB (Yan et al., 2019). A previously published study showed that the well-preserved silages need the numbers of *Lactobacilli* to be at least 10^5^ cfu/g FM of ensiling (Cai et al., 1999c). However, in this study, the numbers of LAB and undesirable microorganisms were lower (10^5^ cfu/g) and higher (10^4^–10^8^ cfu/g) in FM than the required values, which could lead to poor fermentation quality. Additionally, LAB groups also included other species that may have few effects on fermentation. Consequently, the use of LAB additives is necessary for native grass silage; not only the low moisture, WSC content, and LAB counts, but also the growth of undesirable microorganisms was inhibited during the early stages of fermentation.

The DM content was lower in the inoculated groups than in the control in this study. Besides, the WSC content was significantly reduced in inoculated silages compared to the control silage, which agrees with Kleinschmit and Kung (2006), who found that grass silages with inoculant had a lower WSC content than that in the control. The WSC contents were fermented by LAB and transformed into organic acids, ethanol, and carbon dioxide by microorganisms during silage fermentation (Muck et al., 2017; Wang S. et al., 2018). The acid hydrolysis during ensiling also reduced the DM content (Zhao et al., 2018). Therefore, the DM, WSC, NDF, ADF, and pH decreased in inoculated silages. The CP and NH_3_-N contents in control were lower and greater than those in the inoculated silages, reflecting the growth of undesirable microorganisms and the accumulation of NH_3_-N during ensiling (Dong et al., 2020). The higher CP and lower NH_3_-N contents in inoculated silages may be attributed to the lower pH, which inhibits the growth and activities of undesirable microorganisms (Arriola et al., 2011; Heinritz et al., 2012).

The pH value in inoculated silages was lower than that in the control, while the lactic acid in the control was lower than that in L, XM2, and 265 groups. These results may be attributed to a high amount of LA with reducing pH values in the anaerobic environment (Fiyla, 2003). A previous study indicated that a pH of less than 4.20 could inhibit the growth of harmful bacteria and ensure fermentation quality (Wang et al., 2019). The pH value of the control was significantly higher than that of inoculated silages, and the pH value was 4.42. The lower number of LAB and higher numbers of yeasts and aerobic bacteria may be the main reason because the LA and other nutrients used by yeasts, molds, and other aerobic microorganisms produce large quantities of metabolism, resulting in pH increase (Woolford, 1990). In this study, the coliform bacteria and mold were undetectable after 30 days of fermentation, which is in agreement with the report that coliform bacteria and mold were not detected after ensiling for 30 days (Dong et al., 2020). The growth of coliform bacteria and mold inhibited by lower pH was the main reason (Lima et al., 2010; Reich and Kung, 2010).

Amplicon sequencing of bacteria in fresh native grass and silage samples was performed. Good’s coverage values in fresh matter and silages were higher than 99%, suggesting that sequencing could adequately reflect the dynamic change in the bacterial community (Yang et al., 2019). Compared to FM, the diversity and richness were reduced in inoculated silages, especially in inoculated silages, which follow Ogunade et al. (2018), who found that LAB additives could reduce the bacterial diversity due to the increase in the abundance of the predominant genus. The antibacterial activity that affects the composition of different bacteria and the lower pH inhibiting the growth of the desirable bacteria in this study may be the main reason (Bai et al., 2020). Compared to the control, the diversity and species richness of the microbial communities in the LAB-treated silages decreased, which agrees with previous studies that show that the addition of LAB could reduce diversity and richness (Dong et al., 2019).

The PCoA plot showed a clear separation of bacteria in FM and silages, which indicated that ensiling reconstructs the microbial community. These results followed the work of Zeng et al. (2020) that showed that the bacterial communities were distinguished in FM and silages. Besides, compared to the control, the PCoA of LAB-treated silages was also separate, which showed that the additives significantly influenced the microbial community.

This study showed that *Proteobacteria* was the most abundant phylum in FM, comprising more than 80% of the microflora, which agrees with reports that *Proteobacteria* was the dominant phylum in fresh forage (Dong et al., 2019). After 30 days of ensiling, the abundance of *Firmicutes* increased and dominated the fermentation in the native grass silages (80%), and the abundance of *Proteobacteria* decreased. These results were similar to the reports of Yang et al. (2019) and Bai et al. (2020). Compared to the control, the abundance of *Firmicutes* and *Proteobacteria* was significantly increased and lower in L and XM2 groups, whereas no significant differences were observed among control, 265, and 842 groups, which the higher microbial populations of LAB could explain.

This study showed that *Pantoea* was the major facultative aerobic genera in FM. *Pantoea* has been discovered in fresh alfalfa (Ogunade et al., 2018) and soybean (Ni et al., 2017). After 30 days of fermentation, drops in *Pantoea* abundances could be attributed to their high sensitivity to pH decline (Ogunade et al., 2018). *Lactobacillus, Pedicoccus, Weissella*, and *Leuconostoc* are considered as the four most predominant LAB genera responsible for driving lactic fermentation during ensiling (Pang et al., 2012; Ni et al., 2017; Guan et al., 2018; Liu et al., 2019). During ensiling, a significant shift in the bacterial community from *Proteobacteria* to *Firmicutes* could be explained by increased abundance of genera *Lactobacillus*, *Weissella*, and *Pediococcus*, which flourished in the environmental conditions developed during ensiling (Keshri et al., 2019). Therefore, the abundance of *Lactobacillus*, *Enterobacter*, *Pediococcus*, and *Weissella* increased and dominated the silage fermentation among silages (80%). In this study, the LAB-treated silages exhibited an increase in *Lactobacillus* than in the control, which agrees with the findings that LAB-treated silages could increase the abundance of *Lactobacillus* (Liu et al., 2019; Yan et al., 2019; Bai et al., 2020). Additionally, the highest abundance of *Lactobacillus* was found in the XM2 group; strain XM2 isolated from native grass and the higher microbial populations in the XM2 group may be the main reason. *Weissella* was the other predominant microbe in all silages throughout the fermentation and was the early colonizer (Graf et al., 2016; Guan et al., 2018); *Pediococcus* contributed to an initial decline in silage pH, resulting in an anaerobic environment suitable for developing *Lactobacillus* (Yang et al., 2019). The previously published studies showed that *Weissella* was heterofermentative. These two LAB genera could not thrive at a pH environment lower than 4.5 and were thus active only during the early stages of ensiling. The follow-up lactic acid production mainly depends on *Lactobacillus*, which becomes active and thrives as pH decreases (Cai et al., 1998). However, the abundance of *Weissella* was significantly reduced in the 842 group, which could be explained by adding *L. graminis*, which could inhibit the growth of *Weissella*. The abundance of *Pediococcus* significantly increased in the 265 group, which could contribute to the addition of *P. acidilactici*. *Lactobacillus* was enriched in the XM2 group, and *Pediococcus* was enriched in the 265 group, which is in agreement with the addition of LAB in the XM2 and 265 groups.

## Conclusion

The bacterial community of fresh native grass was discovered to be dominated by *Proteobacteria*, *Actinobacteriota*, and *Cyanobacteria*. This study demonstrated that the addition of LAB could influence silage fermentation by reconstructing microbiota. *Lactobacillus* was the dominant genus in the native grass silages, followed by *Enterobacter* and *Pediococcus*. Strain XM2 exhibited the potential possibility to respond to improving silage fermentation in native grass. Further results indicated that strain XM2 could effectively improve the silage quality, and it was proposed to be a potential starter culture for native grass silage.

## Data Availability Statement

The original contributions presented in the study are included in the article/Supplementary Material, further inquiries can be directed to the corresponding author/s.

## Author Contributions

SY: investigation, methodology, visualization, validation, data curation, writing—original draft, and conceptualization. SD: investigation, software, formal analysis, and writing—review and editing. GG: conceptualization, funding acquisition, supervision, and writing—review and editing. TW: project administration and supervision. YJ: conceptualization, funding acquisition, project administration, and supervision. All authors contributed intellectual input and assisted with this study and manuscript.

## Conflict of Interest

The authors declare that the research was conducted in the absence of any commercial or financial relationships that could be construed as a potential conflict of interest.

## Publisher’s Note

All claims expressed in this article are solely those of the authors and do not necessarily represent those of their affiliated organizations, or those of the publisher, the editors and the reviewers. Any product that may be evaluated in this article, or claim that may be made by its manufacturer, is not guaranteed or endorsed by the publisher.

## Footnotes

1
https://www.omicstudio.cn/tool

2
blast.ncbi.nlm.nih.gov
