# Supplementary material for: Microbial Community and Fermentation Characteristics of Native Grass Prepared Without or With Isolated Lactic Acid Bacteria on the Mongolian Plateau
Source: Front Microbiol. 2021 Oct 1;12:731770. doi: 10.3389/fmicb.2021.731770 (PMC8517267; doi:10.3389/fmicb.2021.731770)
Supplement: Supplementary file 1 [file Table_1.docx]

**Supplementary Table 1｜**Alpha diversity of bacterial of native grass and silages after 30 d fermentation.

| Items | Sequences | OTUs | Shannon | Simpson | Chao 1 | Good's coverage (%) |
| --- | --- | --- | --- | --- | --- | --- |
| FM | 51122 | 1017 | 2.249 | 0.370 | 802.784 | 99.677 |
| CON | 42937 | 206 | 1.713 | 0.300 | 171.642 | 99.891 |
| L | 46759 | 336 | 1.415 | 0.479 | 310.070 | 99.821 |
| XM2 | 61669 | 345 | 1.090 | 0.603 | 356.623 | 99.843 |
| 265 | 51626 | 308 | 1.597 | 0.326 | 343.638 | 99.823 |
| 842 | 37032 | 197 | 0.999 | 0.560 | 234.254 | 99.846 |

FM, fresh native grass; CON, control group; L, commercial inoculant group; XM2, strain XM2 group; 265, strain 265 group; 842, strain 842 group.
