# Supplementary material for: Microbial Community and Fermentation Characteristics of Native Grass Prepared Without or With Isolated Lactic Acid Bacteria on the Mongolian Plateau
Source: Front Microbiol. 2021 Oct 1;12:731770. doi: 10.3389/fmicb.2021.731770 (PMC8517267; doi:10.3389/fmicb.2021.731770)
Supplement: Supplementary file 2 [file Table_2.docx]

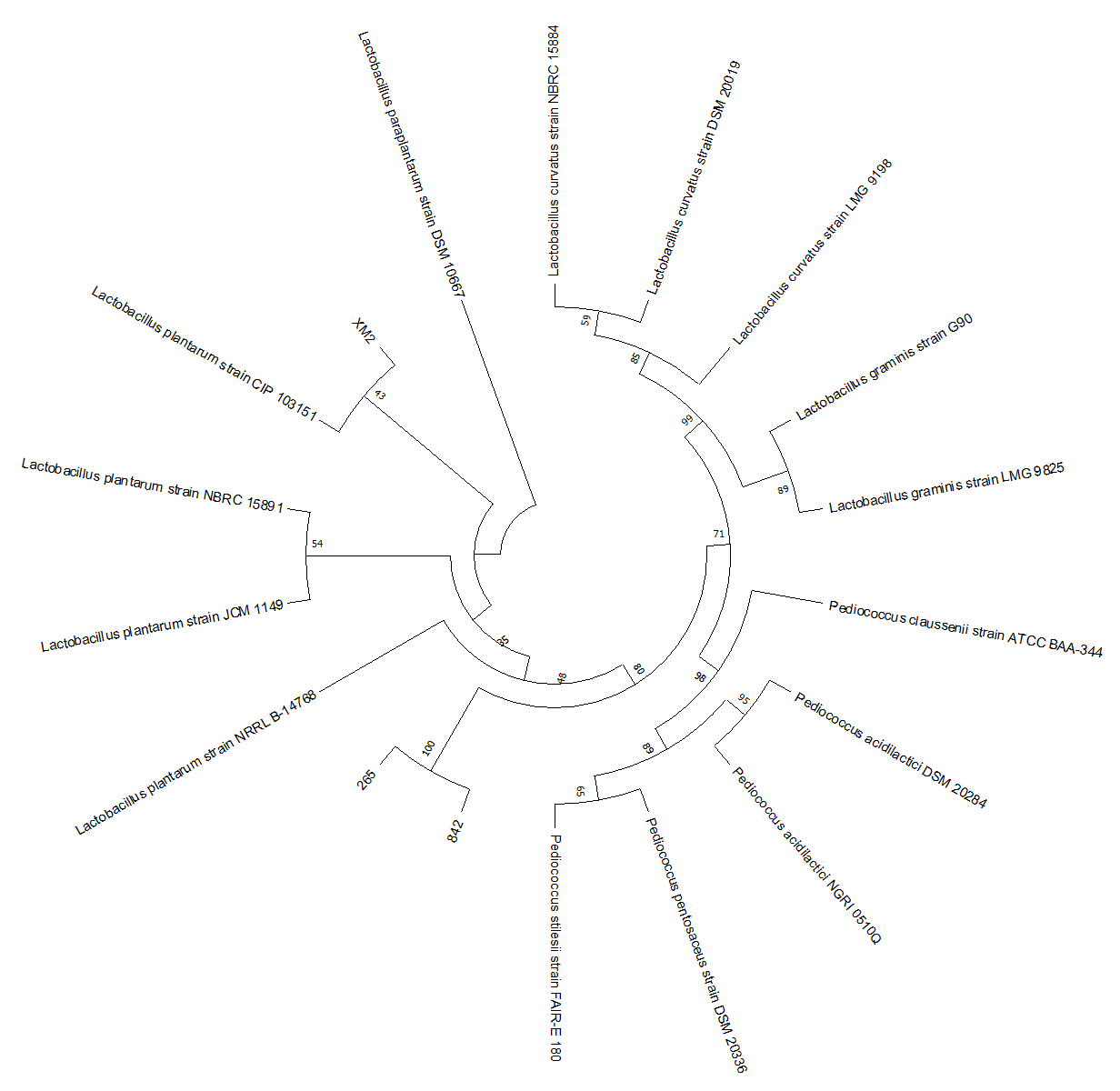


**Supplementary Figure 1｜**Phylogenetic tree of partial 16S rDNA sequences of isolated strains and sequences of identified bacteria in the nucleotide database of GenBank. The bar indicates 1% sequence divergence.
